# Incidental Findings on Whole-Body Computed Tomography Angiography in Patients With Suspected Acute Stroke

**DOI:** 10.7759/cureus.76639

**Published:** 2024-12-30

**Authors:** Takuya Saito, Tatsuhito Ishii, Kazunari Honma, Koichi Watanabe, Masako Sasaki, Yoshiyuki Kondo

**Affiliations:** 1 Neurology, Seirei Hamamatsu General Hospital, Hamamatsu, JPN; 2 Radiology, Seirei Hamamatsu General Hospital, Hamamatsu, JPN

**Keywords:** computed tomography angiography, incidental findings, malignancy, stroke, thrombosis

## Abstract

Introduction

Whole-body computed tomography angiography (CTA) may be useful during cerebral angiography and endovascular treatment (EVT), and identification of thrombi and malignant trunk tumors may be helpful in stroke typing and acute stroke care. Therefore, we aimed to assess the types and prevalence of incidental findings on whole-body CTA in this patient population.

Methods

This single-center, retrospective, observational study included consecutive patients with suspected acute stroke who underwent whole-body CTA in addition to brain CTA between April 2020 and August 2023. Whole-body CTA findings, including vascular lesions, malignancies, and thrombi, were retrospectively collected from medical records and radiology reports. We analyzed data from 290 patients (167 with ischemic stroke, 53 with subarachnoid hemorrhage, 38 with hemorrhagic stroke, and 32 with non-stroke).

Results

The median age was 76.0 years, and the median National Institutes of Health Stroke Scale score on admission was 10. One hundred twenty-three patients (42.4%) underwent digital subtraction angiography (DSA). Of the 38 whole-body CTA findings for patients with suspected acute stroke, 19 were vascular lesions, seven were malignancies, and 12 were thrombi.

Conclusion

These findings show that malignancy and thrombosis, in addition to vascular lesions, can be detected when whole-body CTA is performed in patients with suspected acute stroke.

## Introduction

Computed tomography angiography (CTA) of the head is the gold standard for acute stroke imaging studies [1]. Specifically, carotid evaluation of candidates for endovascular treatment (EVT) may be useful for screening vessel anatomy, carotid artery dissections, stenoses, and occlusions [2]. On the other hand, vascular evaluation of the whole body allows for evaluation of the trunk, in addition to the head, and carotid arteries. As such, whole-body CTA may be useful during cerebral angiography and EVT. Furthermore, the identification of thrombi and malignant trunk tumors may be helpful in stroke typing and acute stroke care. However, reports on the usefulness of whole-body CTA in patients with suspected acute stroke are limited. Therefore, we aimed to assess the types of abnormal findings obtained by whole-body CTA on admission in patients with suspected acute stroke.

## Materials and methods

Study population

The subjects in this study were retrospectively identified from consecutive patients who were suspected of having an acute stroke within 24 hours of symptom onset and admitted to Seirei Hamamatsu General Hospital (Hamamatsu, Shizuoka, Japan) between April 2020 and August 2023. Whole-body CTA was routinely performed in our hospital as a vascular evaluation of the trunk for cerebral angiography in patients suspected of having an acute stroke. We included patients who underwent CTA of the head and whole body upon admission. The exclusion criteria were as follows: 1) patients with suspected stroke more than 24 hours after symptom onset, 2) patients who did not undergo CTA of the head or whole body at admission, and 3) patients under 18 years of age. Clinical and investigative data were prospectively entered into the Seirei Hamamatsu General Hospital Stroke Registry in a standardized fashion by stroke neurologists and research nurses. The severity of neurological deficits was evaluated on admission using the National Institutes of Health Stroke Scale (NIHSS) score [3]. We also collected data on intravenous recombinant tissue plasminogen activator (alteplase), digital subtraction angiography (DSA), or mechanical thrombectomy (MT). The use of alteplase, DSA, or MT was determined by physicians. Alteplase was administered intravenously (0.6 mg/kg according to Japanese guidelines) [4]. The study protocol was approved by the Ethics Committee of Seirei Hamamatsu General Hospital (ID: 4415). The requirement for written informed consent was waived owing to the retrospective nature of the study, and opt-out methods were used.

Computed tomography angiography

All patients underwent CTA of the head and whole body immediately upon arrival in the emergency room. All CTA scans were acquired on a 64-slice multi-detector row CT scanner (OPTIMA CT 660, GE Healthcare) with volumetric acquisition and the following scanning parameters: tube voltage of 120 kV, auto tube current, 0.5-second gantry rotation, and 64×0.625 mm collimation. In all cases, 540 mg/kg of iodinated intravenous contrast medium was administered within 20 seconds using a power injector (dual-shot GX-7, Nemoto), with a scan delay of 20 to 25 seconds after brain CTA with bolus-tracking in the carotid artery. Adding whole-body CTA to head CTA took a few minutes. The CTA scan was performed with a normal dose conforming to the Japan diagnostic reference levels 2020 [5].

Image analysis

The CTA images were evaluated by a neurologist or neurosurgeon in the emergency room and re-evaluated by one or two radiologists the next day. The incidental findings were categorized as vascular lesions, malignancies, or thrombi. Vascular access challenges were examined focusing on points that required attention when performing EVT using a femoral approach. Known malignant lesions were excluded; only malignant lesions that had not previously been noted were included. Lesions highly likely to be benign or those for which malignancy was ruled out after hospitalization were excluded. Thrombosis includes arterial or venous thrombi in major organs and vessels of the trunk. Deep vein thrombosis beyond the femoral vein was not included in the imaging range and could not be evaluated.

Statistical analysis

All statistical analyses were conducted using JMP 13.0 (SAS Institute, Inc.). The collected data were analyzed using descriptive statistics. Continuous variables are expressed as medians and IQRs and categorical variables as percentages.

## Results

After screening 1,632 consecutive patients with suspected acute stroke, we identified 290 patients who underwent CTA of the head and whole body within 24 hours of stroke onset. The median age was 76.0 years (IQR, 65.0-85.0), and the median initial NIHSS score was 10 (IQR, 4-23). There were 258 patients diagnosed with stroke: 167 (57.6%) with ischemic stroke, 38 (13.1%) with intracranial hemorrhage, and 53 (18.3%) with subarachnoid hemorrhage. Thirty-two patients (11.0%) were diagnosed as non-stroke. Fifty-nine (20.0%) patients were treated with alteplase, and 65 (22.4%) underwent MT. The whole-body CTA of 127 patients (43.8%) was read by radiologists (Table 1).

**Table 1:** Patient characteristics IQR, interquartile range; mRS, modified Rankin Scale; NIHSS, National Institutes of Health Stroke Scale; DSA, digital subtraction angiography; MT, mechanical thrombectomy

Variables	n=290
Age, years, median (IQR)	76.0 (65.0-85.0)
Male, n (%)	137 (47.2)
Female, n (%)	153 (52.8)
mRS before onset, median (IQR)	0 (0-2)
Past medical history	
Hypertension, n (%)	137 (47.2)
Dyslipidemia, n (%)	65 (22.4)
Diabetes mellitus, n (%)	48 (16.6)
Atrial fibrillation, n (%)	55 (19.0)
Malignant tumor, n (%)	36 (12.5)
Onset to door time, min, median (IQR)	157 (61-505)
NIHSS score, median (IQR)	10 (3-21)
Stroke classification	
Ischemic stroke, n (%)	167 (57.6)
Intracranial hemorrhage, n (%)	38 (13.1)
Subarachnoid hemorrhage, n (%)	53 (18.3)
Non-stroke, n (%)	32 (11.0)
Alteplase, n (%)	59 (20.0)
MT, n (%)	65 (22.4)
DSA, n (%)	123 (42.4)
Radiologist read, n (%)	127 (43.8)

**Figure 1:**
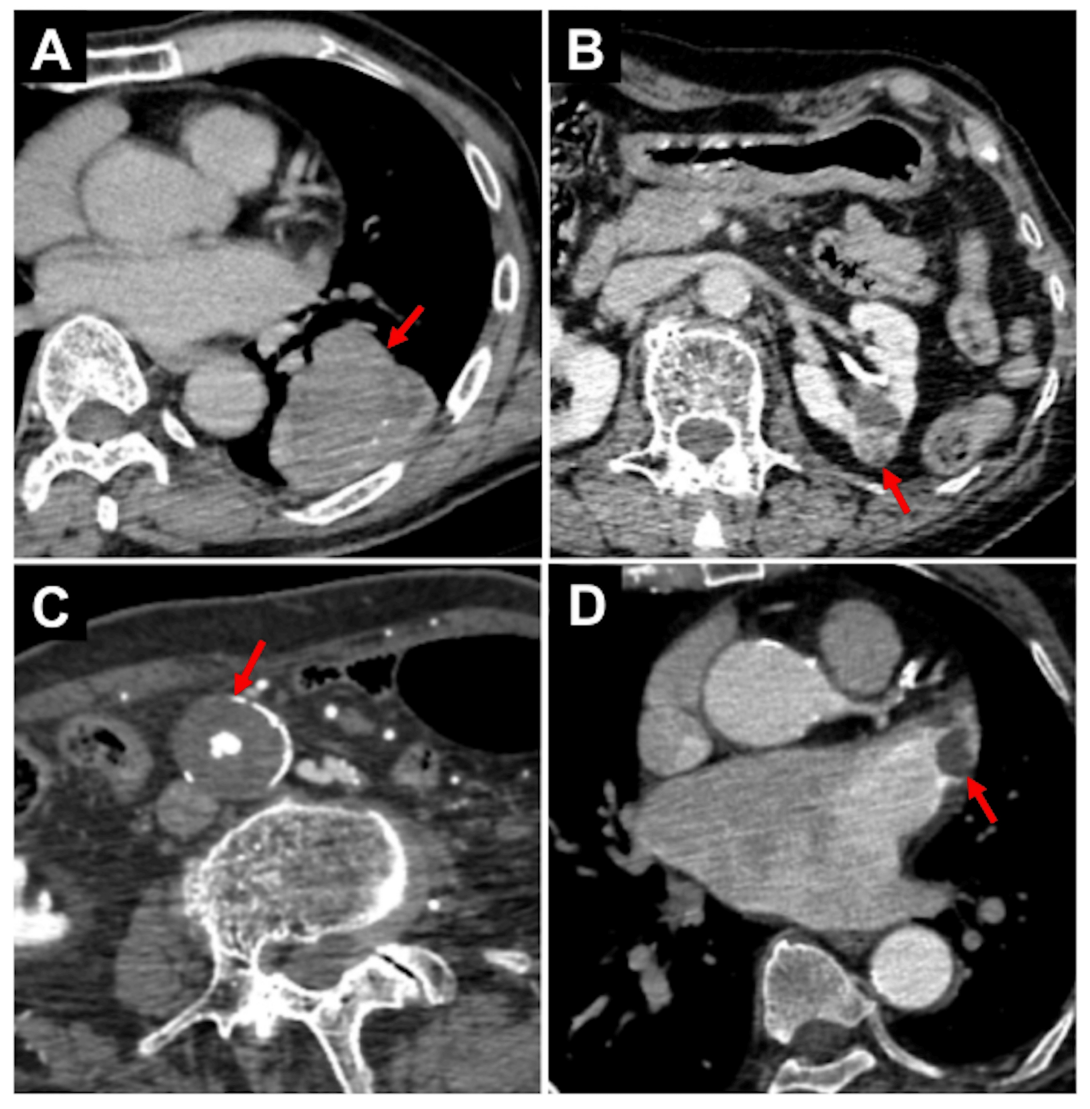
Representative cases of incidental findings on whole-body CTA Arrows indicate the abnormalities in each image. A) A suspected lung cancer lesion in the left lung of a 79-year-old man. B) A suspected kidney cancer lesion in the left kidney of a 91-year-old woman. C) A partially thrombosed abdominal aortic aneurysm in an 83-year-old man. D) A thrombus in the left atrial appendage of an 82-year-old woman. CTA, computed tomography angiography

Overall, 38 incidental findings were identified in the 290 patients. Seven suspected malignant tumor lesions (2.4%) were found: two with lung cancer, two with pancreatic cancer, one with kidney cancer, one with colorectal cancer, and one with choriocarcinoma. Of the seven cases, all were primary tumors, with one case in stage 1, one in stage 2, and five in stage 4. Nineteen vascular access challenges (6.6%) were found: 11 iliac artery or femoral artery lesions (six stenoses, three occlusions, two aneurysms), six descending aorta lesions (four aneurysms, two dissections), four aortic arch lesions (four type A aortic dissections), and one branching from the aortic arch lesion (one aberrant right subclavian artery). Twelve thrombi (4.2%) were found: six intracardiac thrombi, four renal infarctions, two pulmonary embolisms, and one brachial artery occlusion (Figure 1, Table 2). No malignant tumors were found in any of the patients with thrombosis.

**Table 2:** Incidental findings of whole-body CTA CTA, computed tomography angiography

Incidental findings	n=290
Malignant tumor, n (%)	7 (2.4)
Lung cancer, n	2
Pancreatic cancer, n	2
Kidney cancer, n	1
Colorectal cancer, n	1
Choriocarcinoma, n	1
Vascular access challenges, n (%)	19 (6.6)
Iliac artery or femoral artery, n	11
Descending aorta, n	6
Aorta arch, n	4
Branching from the aorta arch, n	1
Thrombosis, n (%)	12 (4.2)
Intracardiac thrombus, n	6
Renal infarction, n	4
Pulmonary embolism, n	2
Brachial artery occlusion, n	1

## Discussion

Our study revealed that whole-body CTA in patients with suspected stroke could be used concomitantly to evaluate malignant lesions, thrombosis, and vascular access challenges. Some patients with suspected stroke have these lesions, and they are an important factor not only in EVT but also in treatment during hospitalization.

Dittrich et al. reported that head and neck CTA after a suspected stroke revealed pulmonary apex lesions in 5.9% of the patients [6]. In patients with suspected acute ischemic stroke who simultaneously underwent cardiac CT and head CT, 9.9% had left atrial appendage thrombus, 1.5% had pulmonary emboli, and 0.3% had suspected breast cancer lesions [7]. Although the CT scans differed in scope and endpoints, the results of the present study did not differ from those of previous studies.

Malignant tumors are more common in patients with stroke than in the general population [8]. Lung and gastrointestinal cancers are particularly common in patients with stroke because risk factors such as smoking are common to stroke and cancer [9]. Furthermore, cancer-induced hypercoagulability has been reported to be associated with ischemic stroke [10]. Comorbid cancer is associated with greater stroke severity, more rapid neurological deterioration, and higher chances of death during hospitalization in patients with ischemic stroke [11]. Patients with stroke may need to be evaluated for cancer complications to understand the pathophysiology and predict outcomes.

The presence of an arterial or venous thrombus may be useful in diagnosing stroke type. For example, an intracardiac thrombus is a high-risk source of cardiac embolism, and venous thrombi such as pulmonary emboli and deep vein thrombosis of the lower extremities are necessary for the diagnosis of paradoxical embolisms. Finn et. al. reported that visceral infarction was more common in patients with cardiac embolism [12].

The identification of vascular access challenges and thrombosis upon admission may improve the clinical outcomes of patients by allowing for better treatment strategies. The clinical picture of acute stroke may not reveal the presence of thrombosis or vascular lesions. However, complications such as pulmonary emboli are associated with poor clinical outcomes [13]. Aortic dissection, brachial artery occlusion, pulmonary embolus, and renal infarction are conditions for which additional treatment, including surgery, should be considered. Stroke in the context of cancer, thrombosis, or vascular disease requires more attention regarding the use of antithrombotic agents.

Our study had several limitations. First, this was a single-center, retrospective investigation, and only eligible patients who underwent whole-body CTA were included. Patients who did not undergo whole-body CTA because of mild symptoms or suspected renal dysfunction were excluded; as such, the results of this study cannot be considered representative of the entire population of patients with suspected stroke. Second, non-assessment lesions were not evaluated, and the reading rate of radiologists was not high in this study. The actual number of lesions that complicate the condition of patients with suspected acute stroke may be considerably higher. Third, we did not evaluate the progression of known malignant lesions. Stroke may occur in association with the progression of malignant lesions. Despite these limitations, this study suggests that whole-body CTA is useful in patients with suspected acute stroke. It should be noted that whole-body CTA involves additional radiation exposure and takes several minutes to perform. Further prospective studies that address these limitations are warranted.

## Conclusions

This study showed that malignancy, thrombosis, and vascular access challenges can be detected using whole-body CTA in patients with suspected acute stroke. Although the frequency of these lesions is limited, they are important findings in the acute management of stroke. Identifying these lesions on admission would improve the quality of acute stroke care.
